# Spatial organization of seismicity and fracture pattern in NE Italy and W Slovenia

**DOI:** 10.1007/s10950-015-9541-9

**Published:** 2015-12-08

**Authors:** G. Bressan, M. Ponton, G. Rossi, S. Urban

**Affiliations:** 1 OGS - Istituto Nazionale di Oceanografia e di Geofisica Sperimentale, Dip. C.R.S., via Treviso 55, 33100 Udine, Italy; 2 Università degli Studi di Trieste – Dip. di Matematica e Geoscienze, Via Weiss, 2-34128 Trieste, Italy; 3 OGS - Istituto Nazionale di Oceanografia e di Geofisica Sperimentale, Dip. C.R.S., Borgo Grotta Gigante, 42/c, 34010 Trieste, Italy

**Keywords:** Seismicity, Geological structures, Crack density, Fractal dimension, PCA analysis

## Abstract

The study focuses on the spatial organization of seismicity and the relation between fracture pattern and earthquakes in the Friuli (north-eastern Italy) and western Slovenia seismic regions. The structural setting is characterized by a complex structure resulting from the superposition of several tectonic phases that generated NW-SE trending Dinaric faults and about E-W trending Alpine faults. The upper crust is characterized by lithological and mechanical heterogeneities. The fractal analysis shows that, in general, the seismicity only partially fills a plane. Only in a few cases, the earthquakes distribute on planar structures. The orientation of planes that fit through the hypocentres shows a different disposition at the two depth intervals analysed. The shallower interval (0–10 km) is characterized by planes with highly variable orientations. The spatial seismicity is investigated in the context of a general damage model, represented by the crack density distribution. The results evidence that the seismicity appears mostly located along sharp transition areas from low crack density to higher crack density, i.e., from zones of low damage to zones of intermediate damage. These zones are characterized by high heterogeneity due to the superposition of different tectonic phases and by the maximum interference between Dinaric and Alpine domains. The orientation of the planes fitting the seismicity at 10–20-km depth appears less dispersed, coinciding with the trend of Dinaric sub-vertical faults in the northern and eastern parts of the study area, and with Alpine low-angle faults in the western and southern parts.

## Introduction

The investigated area is a polyphasic deformational belt, resulting from the superposition of several tectonic phases (Doglioni and Bosellini 1987; Venturini 1991; Ponton 2010) each characterized by a different orientation of the principal axes of stress. This process fragmented the crust into different tectonic domains, with segmentation of the main tectonic lineaments (Slejko et al. 1989). The upper crust (0–10-km depth) is characterized by marked mechanical and lithological heterogeneities, as revealed by the 3D pattern of bulk, shear and Young moduli, obtained with the sequential integrated inversion of tomographic velocity images and gravity data (Bressan et al. 2012). The earthquake spatial distribution appears strictly related to the material heterogeneities because the seismicity is mainly located along the sharp variations of the moduli pattern, in, or close to the high-rigidity zones (Bressan et al. 2012). Except a few cases (e.g., Bajc et al. 2001; Kastelic et al. 2008). most of the spatial pattern of seismicity cannot be resolved on discrete planes, and it seems simplistic to attribute the earthquake locations univocally to a particular individual fault. On the other hand, Cox and Scholz (1988) pointed out that the geometry of faults is complex, with multiple anastomosing strands, and involves large volumes of distributed deformation (fault zone) up to many kilometers.

It is nevertheless true that the seismicity pattern represents an image of the brittle damage of the crust. Therefore, the purpose of the present paper is to analyse the distribution of seismicity within the context of a general damage model, represented by the fracture pattern, and its spatial characteristics.

The fracture pattern is analysed with the crack model of O’Connell and Budiansky (1974) over the area covered by the grid of the sequential integrated inversion of tomographic images and gravity data from Bressan et al. (2012). We point out that the crack pattern is not calculated to perceive the variation of crack density at the scales of the fault zones, but to explain the seismicity pattern within a context of damage. Afterwards, we investigate the spatial distribution of the seismicity, by calculating the fractal dimensions characterizing the spatial pattern of the hypocentres. Finally, the spatial pattern of the seismicity is analysed with the principal component analysis (Ebblin and Michelini 1986; Rossi and Ebblin 1990) to infer the orientations of planes fitting through the earthquake foci.

The fractal dimension and the recognition of the orientations of the planar features fitting the hypocentral data are computed along five deep structural geologic cross sections of the Friuli upper crust (Ponton 2010, 2015) and western Slovenia, to ensure direct comparison with the fault geometries of the tectonic model.

## Tectonic model

**Fig. 1:**
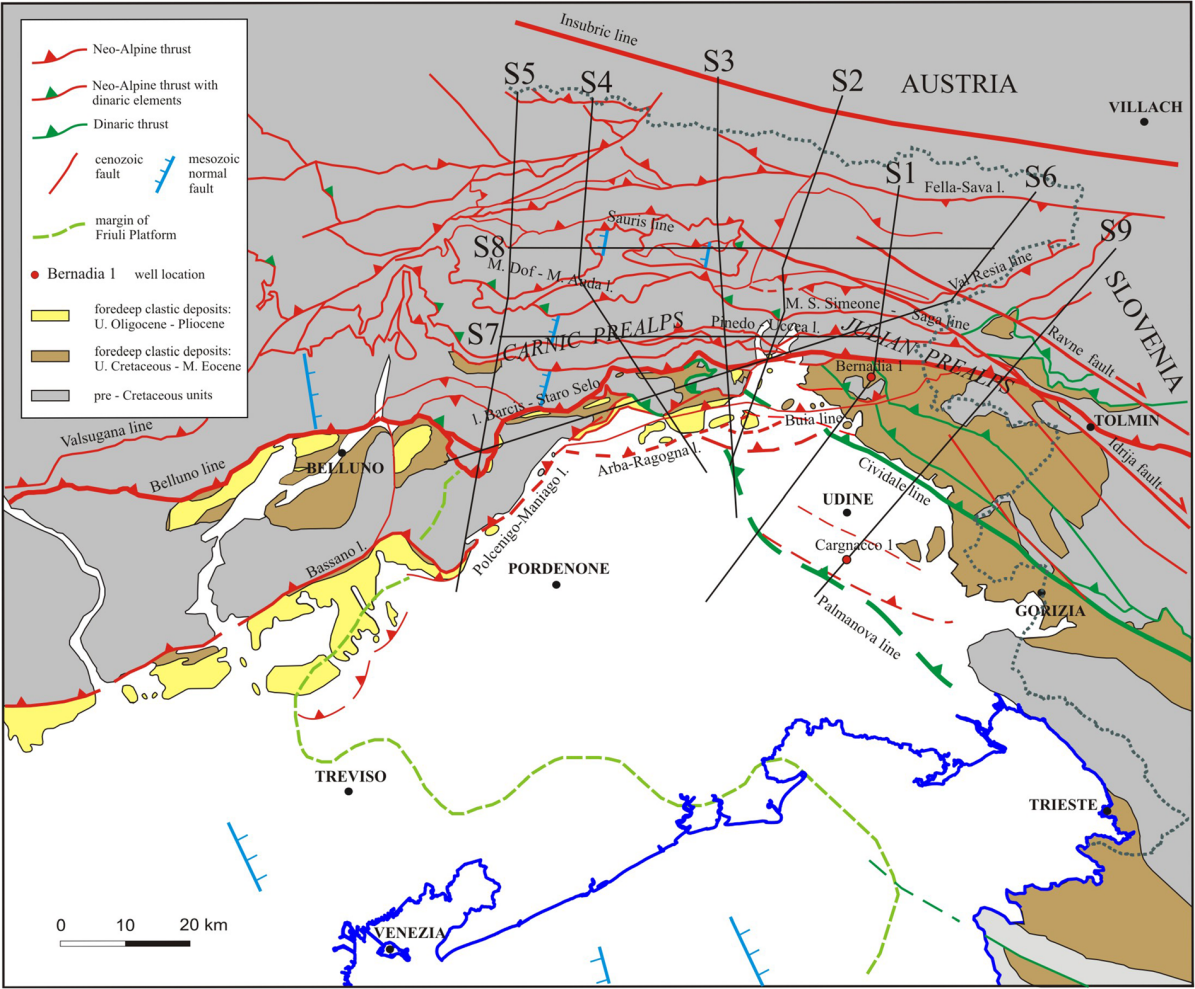
Schematic geological map (modified from Ponton 2010). The main tectonic lineaments are plotted. The traces of the geological cross sections S1, S2, S3, S4, S5, S6, S7, S8 and S9 (Ponton 2010, 2015) are also mapped. The *dots* mark the Italian boundary

The complex tectonic structure (Fig. 1) of the Friuli-Venezia Giulia (north-eastern Italy) and the western Slovenia seismic regions resulted from the progressive counterclockwise rotation and indentation of the Adria microplate with respect to the Eurasian plate (Anderson and Jackson 1987; Mantovani et al. 1996).

The tectonic setting resulted from various tectonic phases. The Mesoalpine (Dinaric) NE-SW compression generated folds and thrust belts that propagated towards southwest during the Upper Cretaceous-Paleogene. The following Neo-Alpine Insubric phase (principal stress direction: NNE-SSW; Chattian-Burdigalian) obliquely cuts the ramps of the Dinaric thrusts, partially reactivating and rotating their fronts in a counterclockwise direction.

The subsequent Valsuganese phase (Serravallian-Tortonian), characterized by NNW-SSE oriented compression, caused a further movement in a counterclockwise direction and generated WSW-ENE oriented thrusts and backthrusts. The last phase, acting from the end of Tortonian to recent times, was characterized by N-S oriented compression. It generated the southernmost thrust system, with orientation varying from NE-SW to E-W and verging from south-east to the south. The NW-SE oriented dextral strike-slip faults, like Idrija Fault and Ravne Fault, were activated in the Julian Alps during this phase.

The stiff Friuli carbonatic platform is inherited from Triassic-Jurassic paleogeographic domains (Carulli et al. 2003; Masetti et al. 2012). Actually, it is present in foreland and central Friuli chain and conditioned the propagation of the shallowest and external overthrusts caused by the crustal underthrusting. The Slovenian Julian carbonatic platform is separated from the Friuli platform by Slovenian Basin, NW-SE oriented (Buser 1989). The N-S, NNE-SSW (western zone) and NW-SE (eastern region) oriented faults, generated by Mesozoic extensional phases along the flanks of the Friuli Platform, were re-activated as strike-slip zones.

**Fig. 2:**
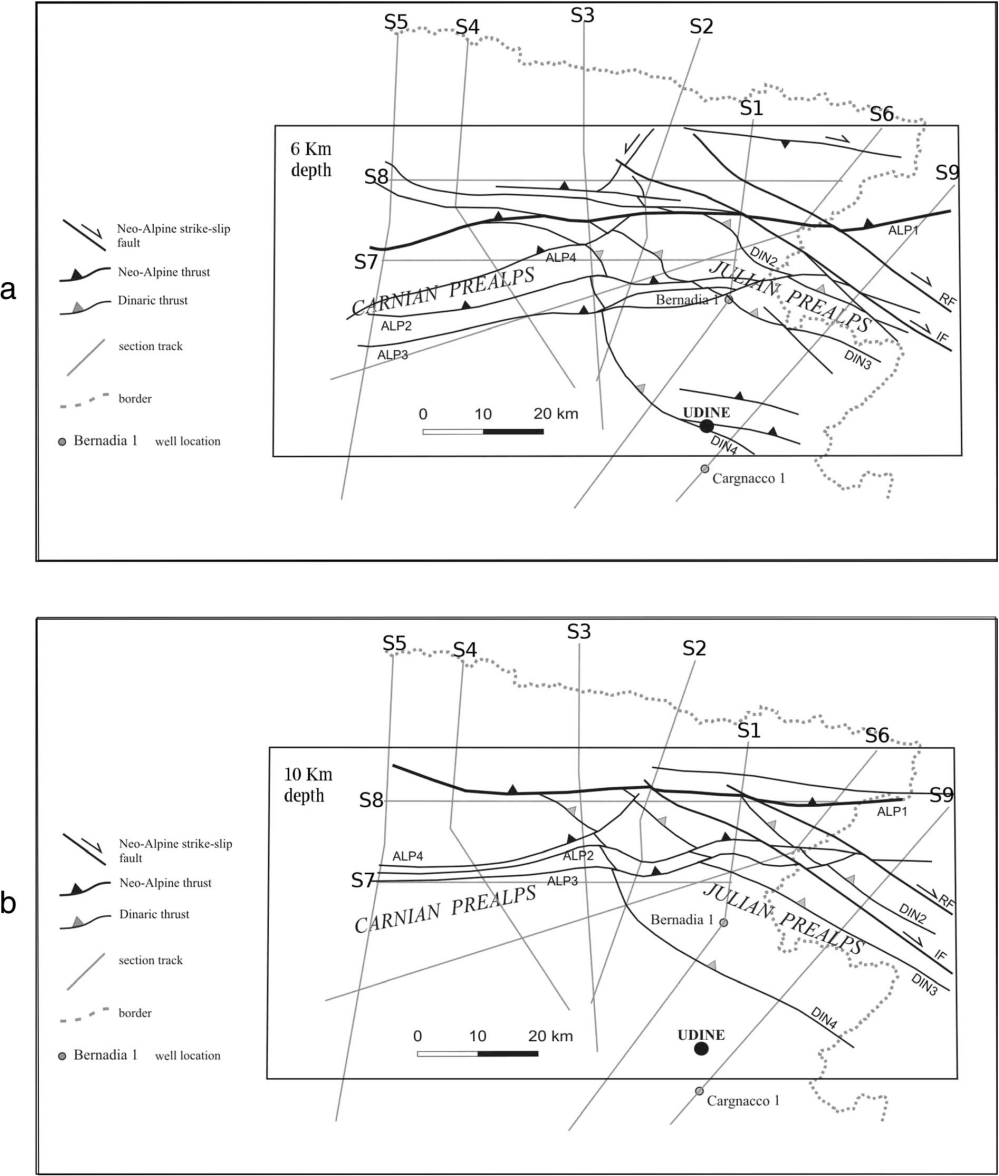
**a** Map of the main tectonic lineaments of Fig. 1, projected at 6-km depth. The traces of the sections, where the fractal geometry and the planar features of seismicity are investigated, are plotted. The bounds of the study area where the fracture pattern is investigated are also marked. **b** Map of the main tectonic lineaments of Fig. 1, projected at 10-km depth. *DIN2* Faedis line, *DIN3* Cividale line, *DIN4* Palmanova Line, *ALP1* Barcis-Staro Selo line, *ALP2* Buia line, *ALP3* Arba-Ragogna line, *ALP4* Polcenigo-Maniago line, *IF* Idrija Fault, *RF* Ravne Fault

The central part of the study area, from Carnic Prealps to Julian Prealps, is characterized by the maximum interference pattern between the NW-SE oriented Dinaric overthrusts and the nearly E-W oriented Alpine thrusts that affect layers mainly from the surface to about 10–12-km depth. Figure 2a, b shows the main tectonic lineaments projected on slices at 6- and 10-km depths, respectively.

The lithostratigraphic sequence ranges from Paleozoic to Quaternary age. The basement is made up of the Paleozoic rocks (mainly sandstones, limestones and locally volcanic deposits) deformed up to anchi-metamorphism (Variscan phases) in some areas (Brime et al. 2008). The basement is NNE dipping, ranging in depth from 8 km in the foreland to 13 km under the chain, where it is considerably shortened by NW-SE and W-E oriented faults (Ponton 2010; Cati et al. 1987). The sedimentary cover overlying the basement is about 10–12 km thick. The stratigraphic sequence consists mainly of Mesozoic carbonatic platform rocks. Thin stratified carbonates and terrigenous successions of basin origin are present elsewhere, particularly in Carnian zone and in western Slovenia with sharp lateral variation with respect to the carbonate platform. Paleogene turbidites, up to 2500 m thick, of the Dinaric foredeep and clastic deposits (Upper Oligocene-Quaternary) of the South-Alpine foredeep terminate the stratigraphic sequence.

**Fig. 3:**
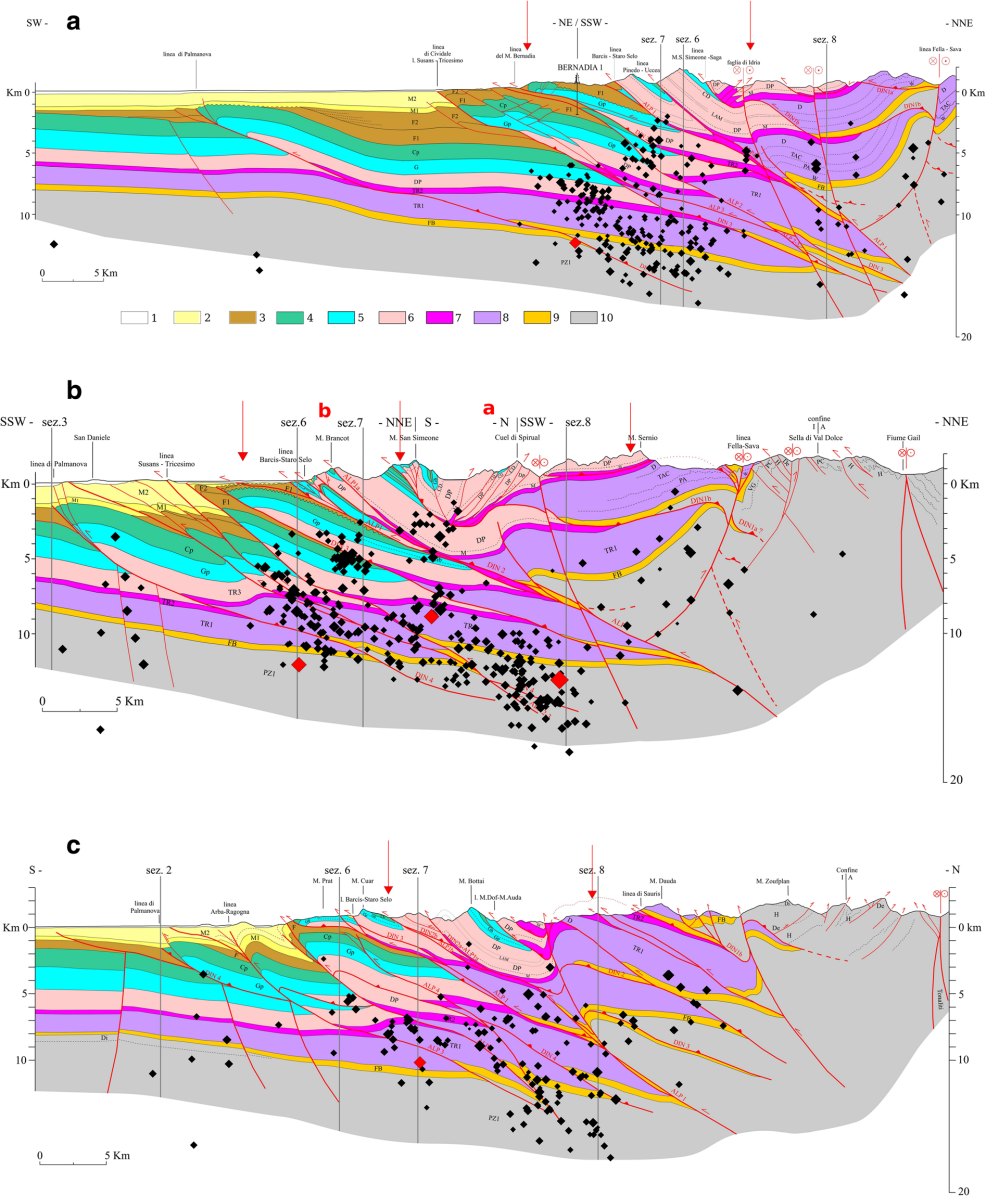

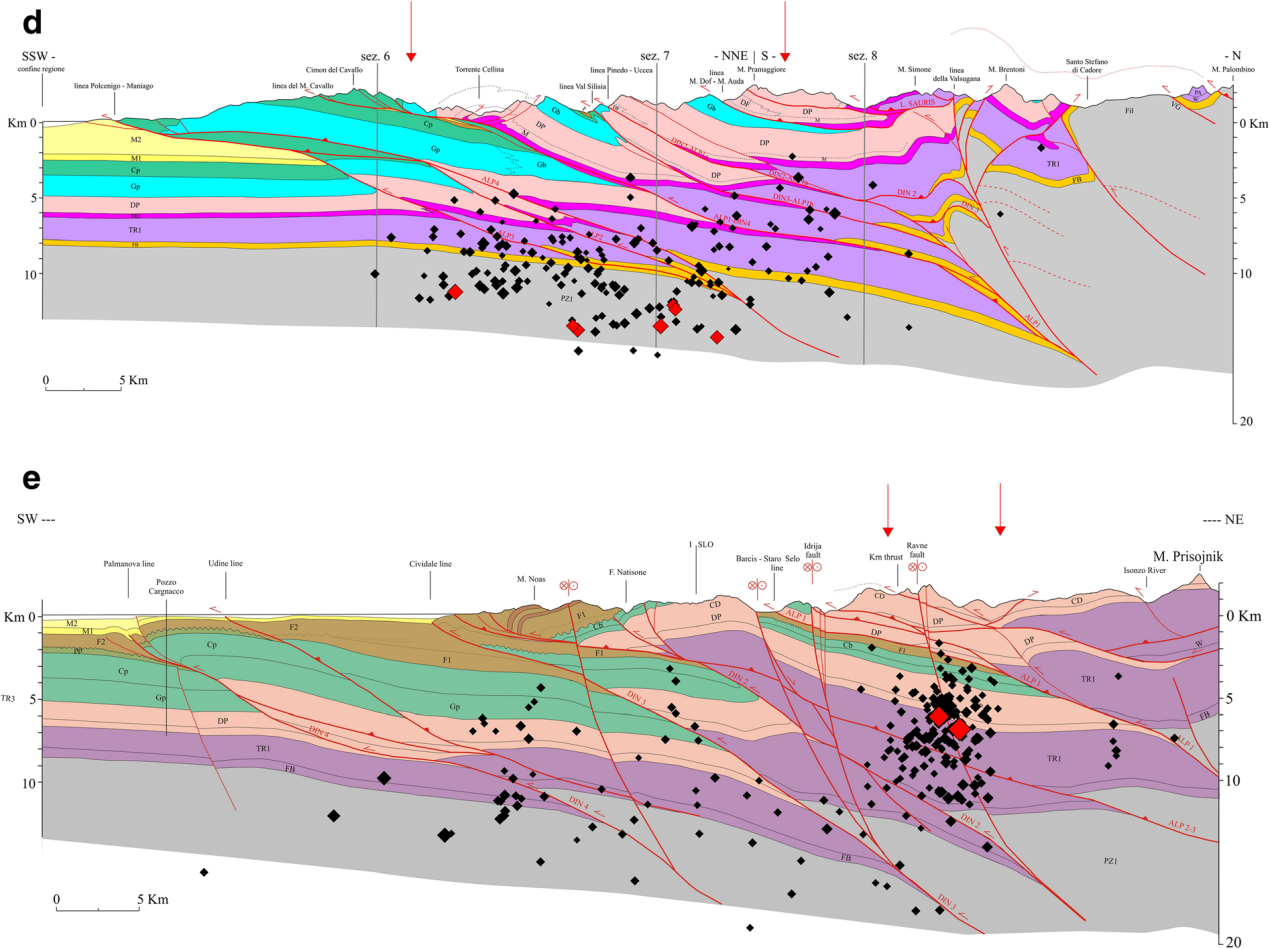
**a** Geological cross section S1. *1* quaternary deposits; *2* molasse deposits (*M1*, *M2* Miocene-Pliocene); *3* flysch deposits and carbonatic levels (*F1*, *F2*, pp; Upper Cretaceous-Eocene); *4* Cretaceous limestones (*Cp* platform, *Cb* basin); *5* Jurassic limestones (*Gp*, *Gb*); *6* Upper Triassic dolomitic rocks (platform and basin) and massive limestones (*DP*, *CD*); *7* Upper Triassic evaporitic, terrigenous and carbonatic rocks (*TR2*); *8* Lower Triassic prevailing limestones (*TR1*); *9* Upper Permian carbonatic, marly and evaporitic rocks (*FB*); *10* Paleozoic terrigenous, carbonatic and locally volcanic deposits (*PZ1*). *Red lines* indicate faults. *Black diamonds* indicate earthquakes. *Red diamonds* indicate seismic events with M_D_ > 3.5. The *vertical arrows* show the zone of the sections where the fractal analysis and the principal component analysis are performed (modified from Ponton 2010, 2015). Also, secondary faults are shown in the section. **b** Geological cross section S2. Symbols and references are as in (**a**). **c** Geological cross section S3. Symbols and references are as in (**a**). **d** Geological cross section S5. Symbols and references are as in (**a**). **e** Geological cross section S9. Symbols and references are as in (**a**)

The geologic cross sections S1, S2, S3 and S5 (Figs. 1 and 3a–d) (Ponton 2010) highlight the geometrical relation between the tectonic structures generated by the different tectonic phases. The sections are about N-S oriented, orthogonal to the Neoalpine faults and oblique to the Dinaric faults. They are characterized by south-verging imbricated tectonic style, with backthrusts in the northern side connected with the uplifting of the Paleozoic basement and interference with the Dinaric structures. The cross sections evidence the intense crustal shortening estimated about 30 km both in N-S and NE-SW directions. The geologic cross section S9 (Figs. 1 and 3e) has been traced in the western Slovenia (Ponton 2015). orthogonal to the NW-SE oriented Dinaric thrusts and the strike-slip faults. The sections were elaborated using original geological survey data and Italian and Slovenian geologic maps (Ponton 2010 and references therein). The geometry of platform bodies, the magnetic basement and volcanic rocks have been derived from a gravimetric-magnetometric survey (Cati et al. 1987). seismic reflection profiles and Bernadia 1 and Cargnacco 1 wells (Merlini et al. 2002; Venturini 2002). The sections were elaborated taking into account also the lateral variation in thickness and facies of the lithostratigraphic units.

To validate our interpretation, we applied cross section balancing technique following well-established rules (e.g. Dahlstrom 1969; Suppe 1983; Woodward et al. 1989; Suppe and Medwedeff 1990). We want to recall that the fault traces along our geological cross sections may be not representative of the real dimension of the fault zones. It is well known, indeed, that the dimension of the fault zone is a function of the activity of the fault itself (e.g. Sibson 1977). This implies that faults with a large displacement are associated with wide fault zones that may reach hundreds of metres. In our case, the area where two fault systems interfere may amplify this process.

The balancing is quite consistent in three dimensions because all sections intersect with each other, despite the complexity of the tectonic structure, resulting from several non-coaxial deformation phases.

The depth projection of the main faults has been drawn using the nine geologic sections of Ponton (2010, 2015). extrapolating the orientation of the faults at the considered depths.

## Seismotectonic aspects

The study area experienced historical destructive earthquakes between 1348 and 1976 (Rovida et al. 2011). with at least eight documented earthquakes of intensity greater than or equal to IX MCS scale. The instrumental seismic activity has been recorded since 1977 by the local seismic network of the OGS - Istituto Nazionale di Oceanografia e Geofisica Sperimentale. It affects mainly the central part of the Friuli-Venezia Giulia region, corresponding to the E-W trending system of south-verging thrusts of Carnian Prealps and Julian Prealps (Fig. 1) and Western Slovenia, related to the Ravne Fault zone (Kastelic et al. 2008). The seismicity is mainly located in the depth range 6–12 km.

The seismotectonic characteristics are heterogeneous. The fault plane solutions are mostly of thrust type, even if with different nodal plane orientations, with a significant number of strike-slip and minor normal faulting events. Several distinct seismotectonic domains have been recognized, and the present stress field is characterized by different stress patterns, with variations in principal stress orientation and stress regime (Bressan et al. 2003). The dominant mode of deformation of the western part of the study area is compressional with maximum compression axis oriented approximately NNW-SSE. The central part of the study area is affected by a general compressional state of stress, with maximum compression axis oriented about N-S. The prevailing mode of deformation in the eastern Friuli and western Slovenia is related to dextral strike-slip motion, with maximum compression axes rotated from NNW-SSE to NNE-SSW. In particular, the area involved by the Ravne Fault zone is characterized by the maximum amount of seismic deformation and seismic strain rate (Bressan and Bragato 2009).

The recorded seismicity is characterized by the occurrence of ten aftershock sequences, triggered by low-moderate earthquakes with coda-duration magnitude M_D_ (Rebez and Renner 1991) ranging from 3.7 to 5.6. Generally, the sequences are spatially and time clustered, alternating with variably long periods of minor seismicity. The completeness magnitude was found to be 2.0 (Gentili et al. 2011).

The most important sequences that struck the area in the last 40 years were triggered by the 1976 May 6 Mw 6.4 earthquake (Slejko et al. 1999). located in the Julian Prealps, and the 1998 April 12 Ms 5.7 earthquake (Bajc et al. 2001). located in western Slovenia. Aoudia et al. (2000) showed that the 1976 earthquake was caused by a fault-related folding, about E-W trending, dipping about 30° to the north. The mainshock rupture evolved from blind to semi-blind faulting, beneath the Neogene sediments. The pattern of aftershocks suggests a westwards propagation of the rupture. The 1998 April 12 earthquake occurred on right lateral strike-slip fault (Bajc et al. 2001). corresponding to the Ravne Fault (Kastelic et al. 2008). The fault rupture was modeled with a sub-vertical rectangular plane, 13 km long and 7 km wide. The aftershocks were triggered on sub-vertical planes, consistent with the orientation of the Ravne Fault.

## Data analysis

The seismic catalogue consists of 2534 earthquakes, occurred from 1988 to 2012, with coda-duration magnitude M_D_ ranging from 1.6 to 5.6.

**Fig. 4:**
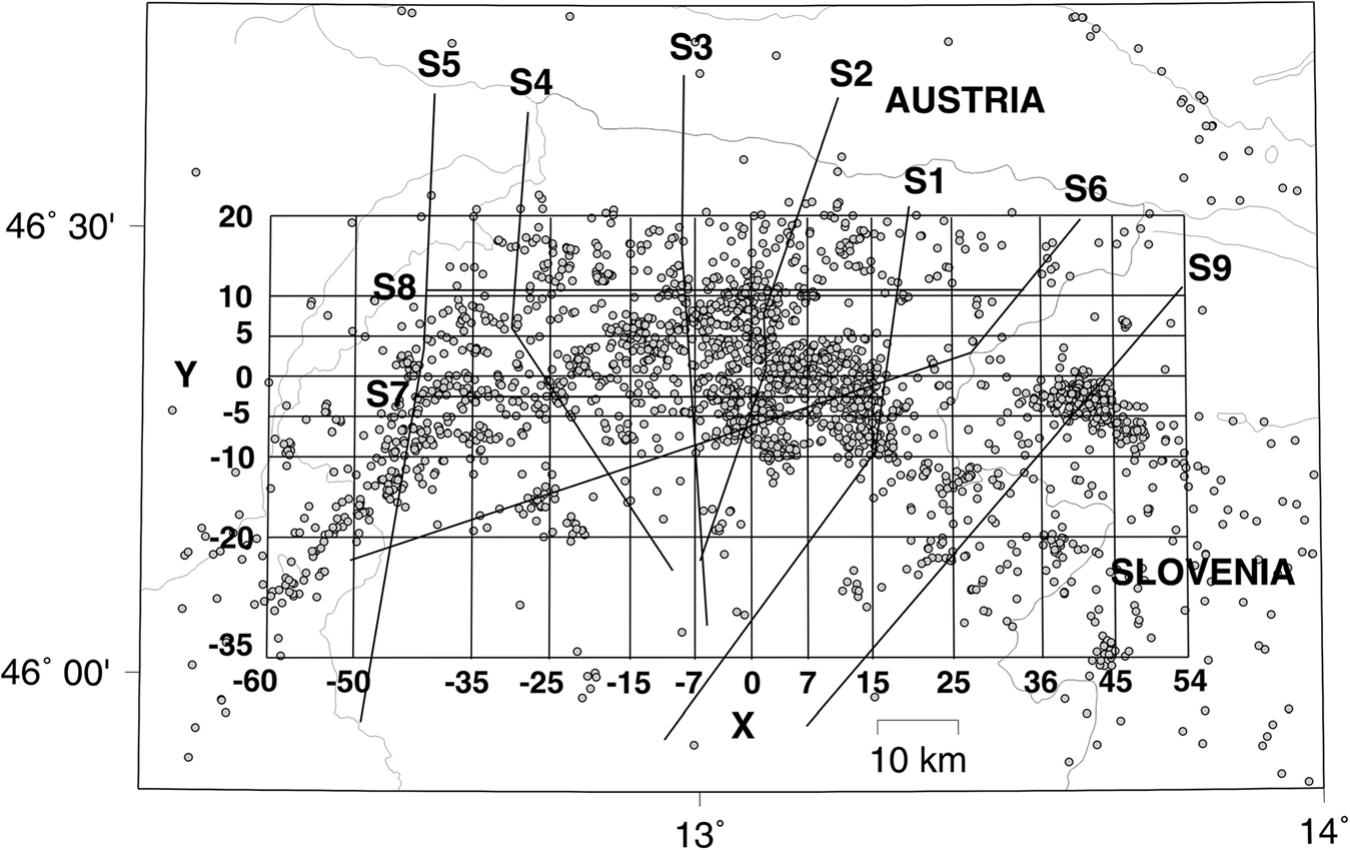
Study area, represented by the grid, where the fracture pattern, the fractal geometry and the planar features of seismicity are investigated. *Circles* indicate earthquakes. The traces of the geological cross sections S1, S2, S3, S4, S5, S6, S7, S8 and S9 (Ponton 2010, 2015) are also plotted

The earthquakes were relocated with the tomographic 3D velocity model of Bressan et al. (2012). consisting of the P-wave and S-wave velocities obtained at the nodes of a grid that is the same grid used in the calculus of crack density and saturation rate (Fig. 4).

**Fig. 5:**
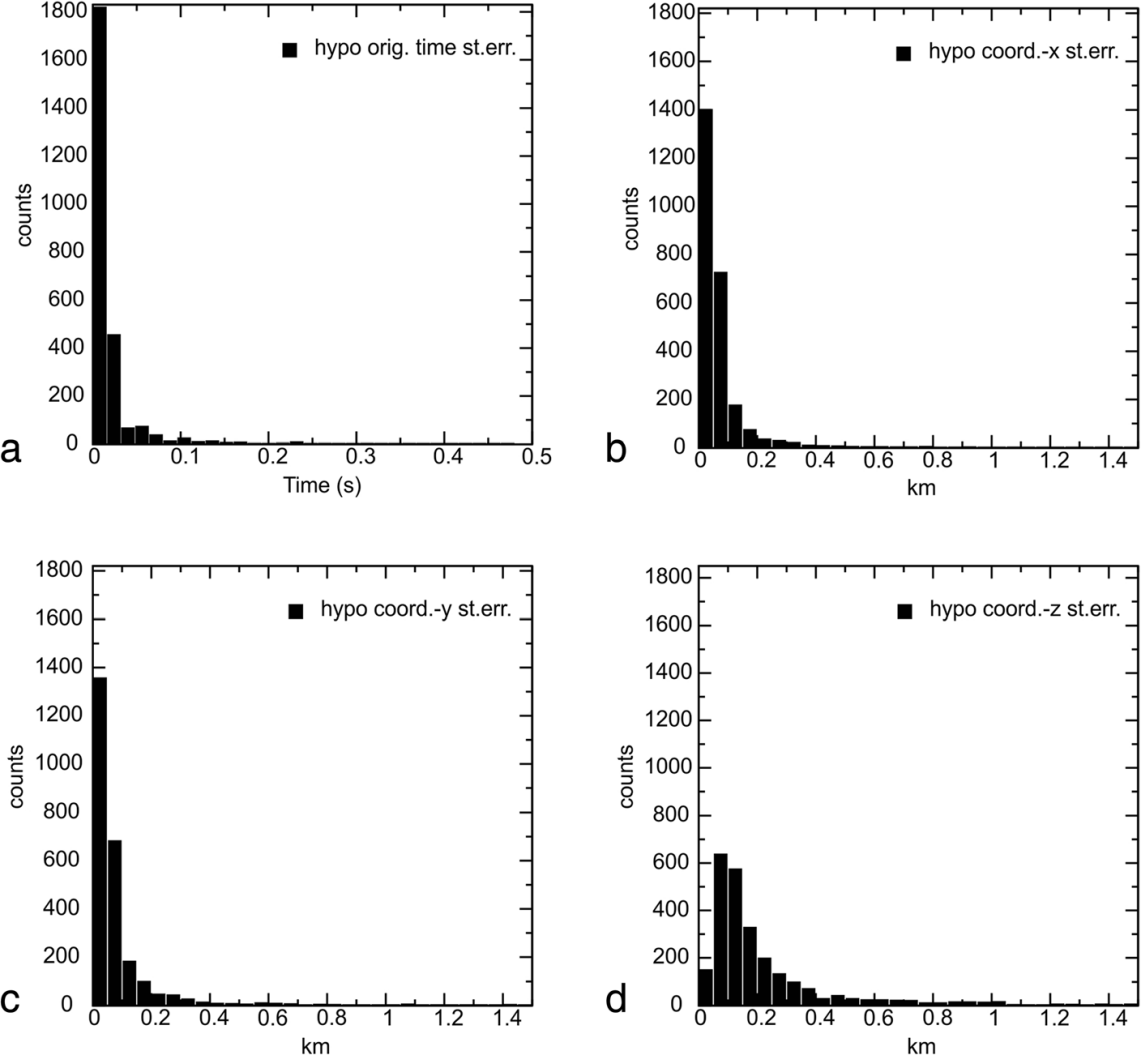
Histogram showing the hypocentral errors versus the number of the relocated earthquakes. **a** Standard errors relative to the origin time. **b** Standard errors relative to *X* coordinates of the grid shown in Fig. 4. **c** Standard errors relative to *Y* coordinates of the grid shown in Fig. 4. **d** Standard errors relative to the depth

The grid is extended 114 km in the west-east direction (W-E grid nodes at *X* = −60, −50, −35, −25, −15, −7, 0, 7, 15, 25, 36, 45 and 54 km) and 55 km in the north-south direction (S-N grid nodes at *Y* = −35, −20, −10, −5, 0, 5, 10 and 20 km). The depth grid spacing is *Z* = 0, 2, 4, 6, 8, 10, 12, 15 and 22 km. The Earth’s topography was included in the model. The standard errors relative to the hypocentral coordinates are shown in Fig. 5a–d. The mean of standard errors relative to the hypocentral coordinates are 0.26 km (z-depth) with standard deviation of the errors 0.56 km, 0.09 km (*X* coordinates) with standard deviation of the errors 0.26 km and 0.1 km (*Y* coordinates) with standard deviation of the errors 0.28 km. The errors are considerably smaller than the distances among the main faults traced in the geological cross sections.

The catalogue is declustered from the aftershock sequences using the results of Gentili and Bressan (2008) since the present study regards a spatial analysis of the seismicity to ensure direct comparison with the fault geometries.

According to Scholz (1990). aftershocks are considered to be a secondary process induced by the dynamic loading of the mainshock that causes a readjustment of stress in the volume surrounding the mainshock fault zone. Therefore, the aftershocks result from the interaction between the induced stress field of the mainshock and the mechanical heterogeneities of the medium, which causes different loading states and stress rotation. Under these conditions, also, the variation in normal stress can be effective as an increase in shear stress in promoting failure. The aftershocks occur on favourably oriented pre-existing faults and discontinuity planes. The discontinuity planes can also be the boundaries between mechanically different rocks because the interface separating media with different elastic moduli is energetically favourable for localization of fractures. The mechanical rock discontinuities at the layer interfaces cause rotation of the stress field due to the mainshock, depending on the contrast in rock elastic moduli, favouring failure.

The spatial distribution of the 1998 aftershocks defines vertical planar features and splays consistent with the orientation of the strike-slip Ravne Fault (Bressan et al. 2009). However, off-fault aftershocks were observed (Bressan et al. 2007) in cases of dip-slip fault. The aftershocks form trails delineating sub-vertical planes above and with different orientations in respect to the mainshock fault plane, not related to any known fault.

The fractal analysis and the search for possible planar features on which the seismic events distribute (principal component analysis, or PCA) are performed along five structural geologic cross sections for a direct comparison with the tectonic structure of the upper crust. The seismicity is analysed on bands extended laterally ±5 km for sections S1 and S5, ±3.5 km for sections S2 and S3 and ±2.5 km for section S9 (Fig. 3a–e). The analysis is performed for each resulting “polygon” on two depth intervals, from the surface to 10-km depth and from 10- to 20-km depths, respectively. Ten kilometres is the approximate depth level of the horizon detachment of the Mesozoic cover from the Paleozoic geologic units, characterized by a different dominant tectonic pattern. The section S2 (Fig. 3b) is divided into two parts (a, b) because of the different tectonic structure. The part a is characterized by a high-interference pattern between the Dinaric and Alpine thrusts while south-verging Alpine thrusts mainly characterize the part b.

We calculated the maximum interval in latitude, longitude and depth for each subset, since both fractal and PCA analyses deal with the spatial coordinates of the hypocentres, to be sure that the results are not biased by a too large discrepancy of one particular dimension with respect to the others. The intervals for the three dimensions vary from a minimum of 3 km to a maximum of 21 km, with a maximum ratio of 3 between them (S2b, the deepest part; S5, the shallowest part).

## Crack density and saturation rate

O’Connell and Budiansky (1974) formulated a model (hereafter called OB74 model) that relates the variation in seismic velocities, Poisson’s ratio and elastic moduli to the crack density and saturation rate of rocks. The model is based on the assumption that flat circular cracks are randomly oriented and are filled with fluids. O’Connell and Budiansky (1974) defined the crack density parameter as

1 $$ \varepsilon =N\left\langle {a}^3\right\rangle $$

where *N* is the number of microcracks per unit volume and 〈*a*〉 is the average radius of circular cracks. Assuming that *N*_1_ cracks per unit volume are dry, and that *N*_2_ = *N* − *N*_1_ cracks are saturated, the OB74 model defined the saturation rate as

2 $$ \xi =\frac{N_2}{N} $$

The relations of the OB74 model are effectively summarized by Zhao and Mizuno (1999). among which we use the following to calculate the crack density and the saturation rate:

3 $$ \frac{\overline{E}}{E}=1-\frac{16}{45}\left(1-\overline{\upsilon^2}\right)\;\left[3\left(1-\xi \right) + \frac{4}{2-\overline{\upsilon}}\right]\varepsilon $$

4 $$ \frac{\overline{G}}{G}=1-\frac{32}{45}\left(1-\overline{\upsilon}\right)\;\left[1-\xi + \frac{3}{2-\overline{\upsilon}}\right]\varepsilon $$

where *Ē*, $\overline{G}$ and $\overline{\upsilon}$ are the Young’s modulus, shear modulus and Poisson’s ratio of the cracked rocks, respectively. *E*, *G* and *υ* are the same parameters of the uncracked rocks. *ε* and *ξ* are the crack density and the saturation rate, respectively.

Zhao and Mizuno (1999) applied the OB74 theory to Vp, Vs and Poisson’s ratio obtained from seismic tomography in the 1995 Kobe earthquake region, assuming that uncracked rocks have the maximum seismic velocity at each depth in the study area. However, Zhao and Mizuno (1999) highlighted a crucial aspect in applying the OB74 model to tomographic results. Temperature and lithological changes affect seismic velocities, and their influence on applying the OB74 model is not clear. In particular, Zhao and Mizuno (1999) pointed out that the method could not be applied to areas where high temperatures are the main cause of the velocity variations.

The seismic properties of the most representative lithologies of the Friuli upper crust were investigated with laboratory measurements (Faccenda et al. 2007). The rock types selected for laboratory measurements as representative of the sedimentary cover of the studied area constitute roughly 90 % of the total volume of rocks (Faccenda et al. 2007). The seismic intrinsic properties were measured at a pressure up to 450 MPa and used to calculate the elastic parameters. The effect of porosity and pore geometry is negligible or null at confining pressures well above the corresponding pore and microfracture closing level (150–200 MPa).

We apply the OB74 model to the Friuli area, defined by the grid of 3D seismo-gravity integrated inversion (Fig. 4) of Bressan et al. (2012). introducing the following novelties in the model formulated by Zhao and Mizuno (1999).

The lithotypes are assigned at each node of the grid according to the geologic and structural model of the Friuli area outlined by Ponton (2010, 2015). where the geometries and thickness of the sedimentary units are reconstructed with the geological cross sections mapped in Fig. 1. The values of the elastic moduli of the upper crust obtained from the seismo-gravity integrated inversion (Bressan et al. 2012) at the grid nodes are assumed characterizing the cracked rocks. The elastic moduli of the uncracked rocks are represented by the values of the elastic moduli obtained from laboratory measurements at 400 MPa confining pressure (Table 4 in Faccenda et al. 2007).

We exclude effects due to high temperatures in our study area because uniform heat flow generally characterizes the geothermal environment, with values in the range 50–60 mWm^−2^ (Cataldi et al. 1995).

**Fig. 6:**
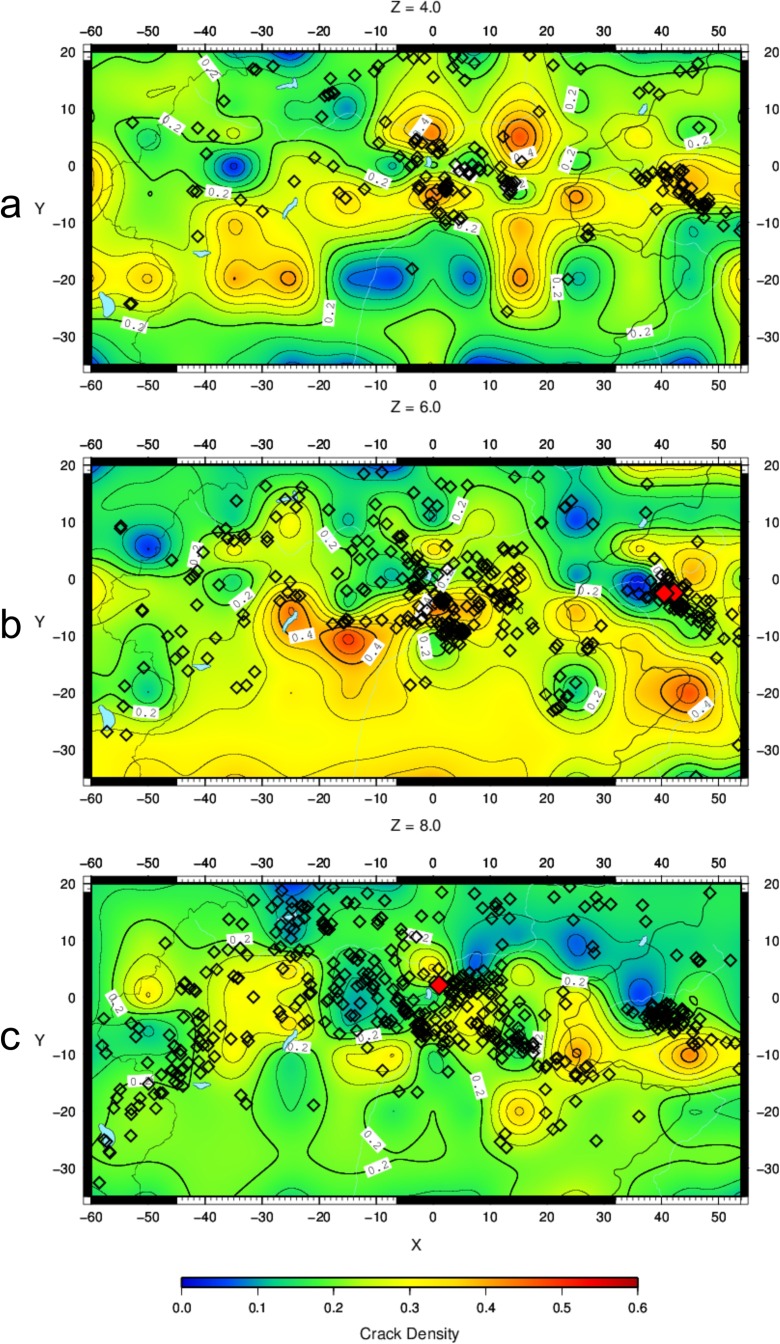
Crack density pattern at 4- (**a**), 6- (**b**) and 8-km (**c**) depths. *Diamond symbols* represent the earthquake locations. The seismicity is plotted within the depth intervals 3–5 km (**a**), 5–7 km (**b**) and 7–9 km (**c**). *Red diamonds* indicate seismic events with M_D_ > 3.5

**Fig. 7:**
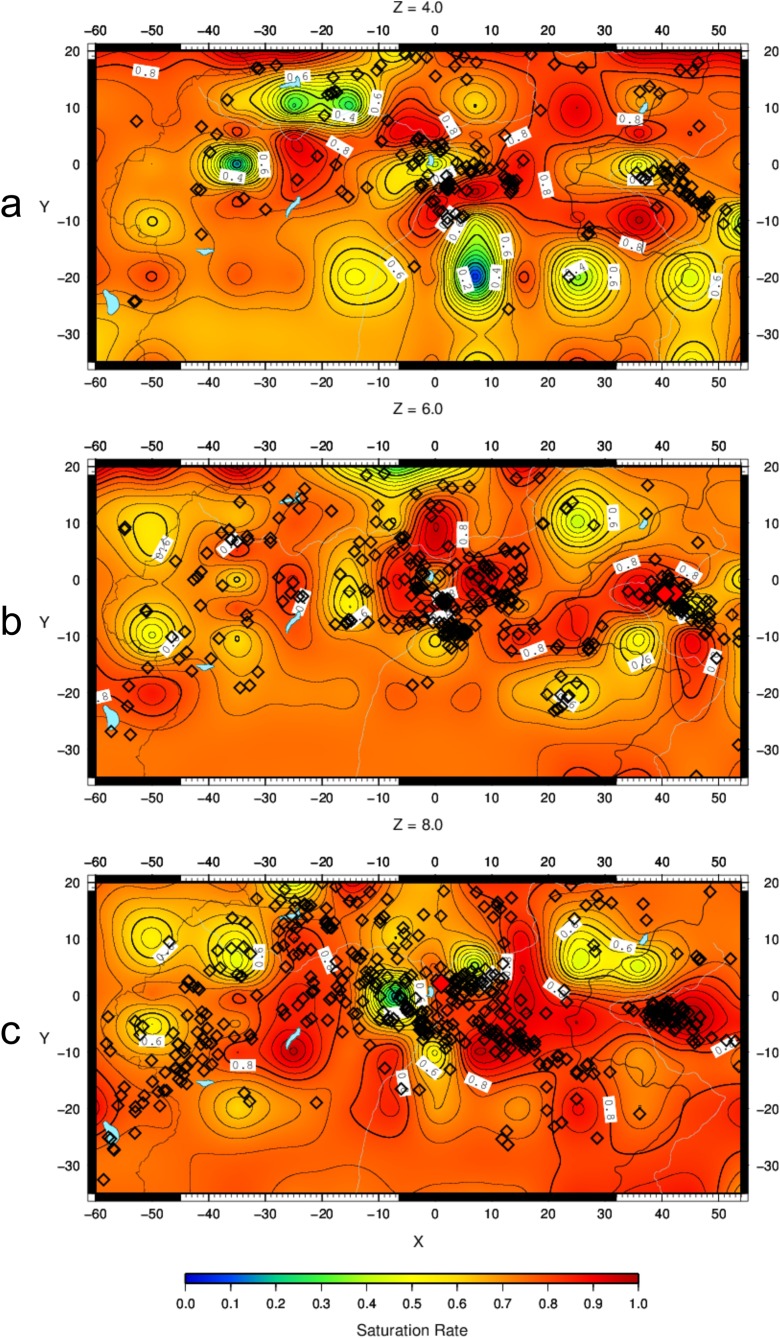
Saturation rate pattern at 4- (**a**), 6- (**b**) and 8-km (**c**) depths. *Diamond symbols* represent the earthquake locations. The seismicity is plotted within the depth intervals 3–5 km (**a**), 5–7 km (**b**) and 7–9 km (**c**). *Red diamonds* indicate seismic events with M_D_ > 3.5

The crack density *ε* (Fig. 6a–c) and the saturation rate *ξ* (Fig. 7a–c) have been calculated on slices at 4-, 6- and 8-km depths, where tomographic images were obtained with good resolution (Bressan et al. 2012). The seismicity is plotted within the depth intervals 3–5, 5–7 and 7–9 km, respectively.

The patterns of *ε* and *ξ* are characterized by high heterogeneity and spots with sharp spatial variation. This pattern reflects the contrast of the Carnian and the Julian Prealps (Fig. 1)—characterized by dolomitic limestones and stiff Triassic limestones—with molasse deposits and less stiff limestones in the foreland. The spots with a sharp spatial variations between analysed depth intervals are due to the repetition of the geological formations caused by overthrusting (Fig. 3a–e) with alternation of high-stiffness rocks (pertaining to Triassic dolomitic limestones) and lower-stiffness rocks (pertaining to less stiff Jurassic and Mesozoic limestones). Moreover, zones with variable density cracks can be present in the same geological formation. The seismicity appears mostly located along the sharp transition from zones of low crack density to areas of intermediate crack density. The saturation rate *ξ* is greater than 0.5 for most of the study area, but no distinctive pattern can be observed in relation to the crack density distribution. The seismicity appears mainly located in areas characterized by high saturation rate.

The concept of damage has been introduced in rock mechanics (Kachanov 1986) to describe the changing state of the structure of the material as it is strained and it considers a progressive deterioration of the physical and mechanical properties under loading stress. In the literature (e.g. Kemeny and Cook 1986). many damage models of rocks have been proposed, all related to crack density, because of a progressive degradation of the elastic moduli. The damage *D* is expressed as a function of the effective elastic modulus *E*_*eff*_ of a reference material and elastic modulus *E*_*D*_ of the damaged material with a scalar relationship:

5 $$ {E}_D=\left( 1-D\right){E}_{eff} $$

As before, we estimate the damage pattern of the study area assuming as effective elastic modulus of a reference material (*E*_*eff*_) the shear modulus *G*_*eff*_ obtained by the laboratory measurements at 400 MPa confining pressure (Table 4 in Faccenda et al. 2007) and as elastic modulus of damaged materials (*E*_*D*_) the shear modulus *G*_*D*_ obtained from the sequential integrated inversion of Bressan et al. (2012):

6 $$ {G}_D=\left(1-D\right){G}_{eff} $$

**Fig. 8:**
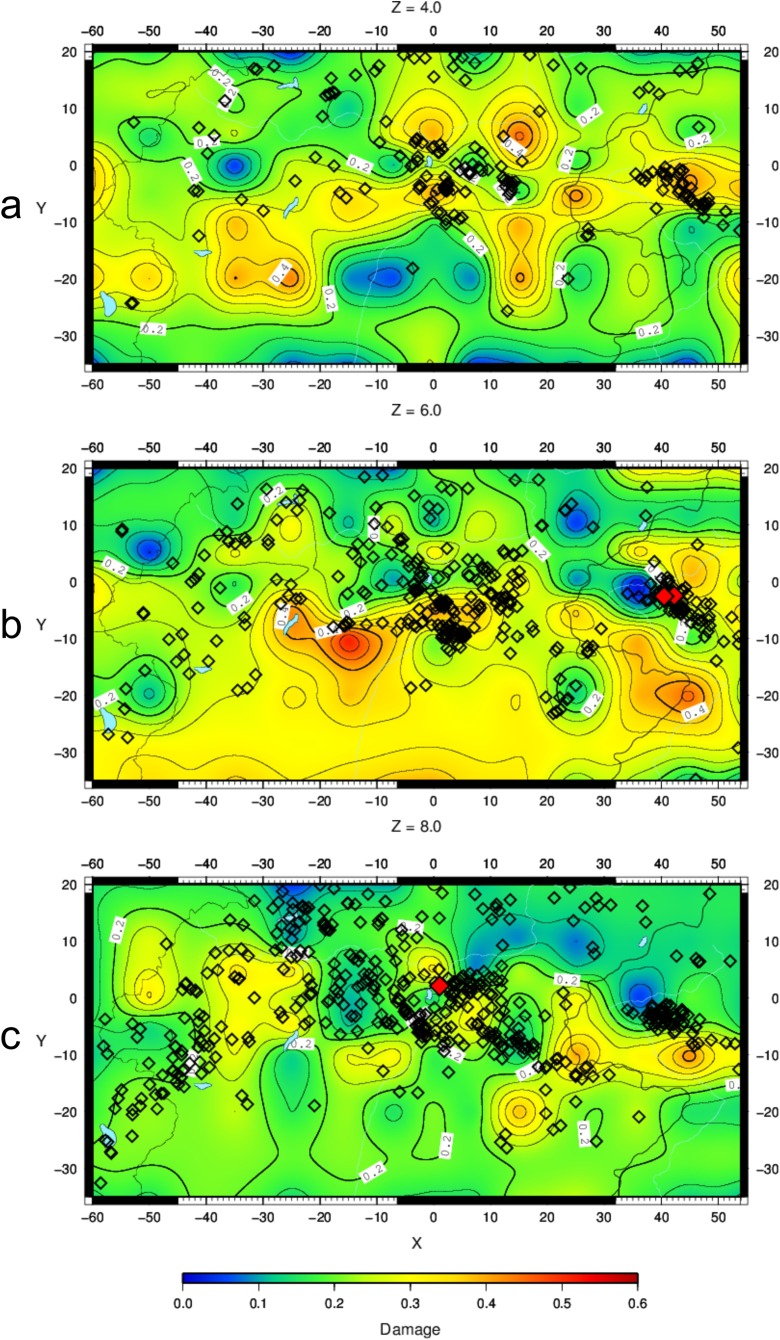
Damage pattern at 4- (**a**), 6- (**b**) and 8-km (**c**) depths. *Diamond symbols* represent the earthquake locations. The seismicity is plotted within the depth intervals 3–5 km (**a**), 5–7 km (**b**) and 7–9 km (**c**). *Red diamonds* indicate seismic events with M_D_ > 3.5

The pattern of damage (Fig. 8a–c) is very similar to the crack density pattern obtained with the OB74 model (Fig. 6a–c). The same comments about heterogeneity and spots with a sharp spatial variation of the crack density pattern can be applied to the damage pattern. The seismicity appears mostly located along the sharp transition from zones of low damage to areas of intermediate damage.

## Fractal analysis

The appeal of fractal geometry and fractal analysis is the recognition of the self-similarity of many natural phenomena and objects, implying the possibility of extending laboratory observations to larger scales. Moreover, the fractal analysis enables to quantify with a single number (namely, the fractal dimension) qualities as roughness, irregularity, clustering tendency, otherwise described only qualitatively. Not an integer number, fractal dimension *D* fills the gaps between the Euclidean dimensions describing a point (0), line (1), a plane (2) and a sphere (3). Hence, it characterizes the spatial distributions intermediate between the extremes described by the Euclidean dimensions allowing, for example, quantifying the tendency of lines to fill a plane with values of *D* fractional and comprised between 1 and 2; the nearer to the latter, the denser is the plane coverage (Mandelbrot 1977; Kagan 1991).

Many authors recognized seismicity and the fracturing process as being fractal since the early beginning of this quantitative approach to the study of nature (among the others, Mandelbrot 1977; Kagan and Knopoff 1980; Kagan 1982; Sadovsky et al. 1984; Turcotte 1986; Hirata et al. 1987; Okubo and Aki 1987; Yamashita and Knopoff 1987; 1989; Hirata 1989). Fractal distribution of earthquakes and acoustic emissions have been tested in time and space while the Gutenberg–Richter and Omori’s power laws provide quantities analogous to the fractal dimensions related to energy. The tight relationship between spatial and time distribution of the seismic sequences is at the basis of the space-time combined correlation integral of Tosi et al. (2008). The generalized fractal relation for the earthquake number proposed by Chelidze (1993) depends on space, time and even energy.

Two kinds of algorithms can be applied to estimate the generalized fractal dimension: the first group is represented by the fixed-size ones, based on the scaling of mass with size for fixed-sized balls or grids. The fixed-mass methods represent the second category, based on fixed-mass balls, more suitable for studying multifractals (Theiler 1990; Kamer et al. 2010).

The correlation integral method (Mandelbrot 1977; Grassberger 1983) gained the primacy among the fixed-size algorithms used for retrieving the fractal dimension of a data set (as, e.g., box-counting method). This is because it is less sensitive to the data number and shape of the point spatial distribution and, hence, more suitable to analyse small data sets, or limited regions (Hirata et al. 1987; Rossi 1990; Theiler 1990; Molchan and Kronrod 2005; Kagan 2007).

According to this method, the fractal dimension *D*_*c*_ is

7 $$ {D}_c=\underset{L\to 0}{ \lim}\frac{ \log C(L)}{ \log (L)} $$

while

8 $$ C(L)=\frac{n}{N} $$

where *n* is the number of couples of points separated by a distance less than *L*, progressively reduced, and *N* is the total number of points. Hence, *D*_*c*_ is the slope of the curve of *C*(*L*) versus *L* in a bi-logarithmic diagram.

The problem of the reliability of the correlation integral estimate in the case of small data sets has been often addressed (e.g. Smith 1988). Havstad and Ehlers (1989) demonstrated that small data sets can also be of use. Eneva (1996) underlined the fact that the correlation integral saturates at large distances while this effect is not present at small distances above the location error radius. Theiler (1986) proposed an algorithm that avoids large distances in the correlation integral calculus, by applying the analysis to subsets of the whole data set. Our choice of performing the analysis within subsets of the catalogue centred on the geological sections and extended laterally lays in these guidelines. Moreover, we decided to evaluate the data sets in three categories (A, B, C) according to three criteria:

1. Order of magnitude of the inter-distances;
2. Constancy of the fractal dimension by using a smaller event number;
3. Limited deviations from the linearity in the log-log plots.

Hence, we evaluate as quality A the data sets with distances distributed on at least two orders of magnitude. In fact, the limitation of the region considered and the prevailing thrust mechanism imply smaller distances between the hypocentres, compared to other catalogues, with different dominant mechanisms and spatial extension (Márquez-Rámirez et al. 2012). Data sets of quality B show distances distributed over less than two orders of magnitude, but with a constant value of the fractal dimension, also using a smaller event number. Data sets of quality C do not maintain the same value diminishing the event number and show relevant deviations from the linearity on the log-log plot.

### Seismic data fractal analysis

Fractal methods were already applied to the study of the seismicity spatial distribution in the study region. In particular, the fractal dimension variations over time were analysed by using two temporal windows of 30 days and 30 shocks, respectively (Rossi 1990, 1994). The analysis evidenced changes of spatial clustering preceding and following moderate earthquakes in the region, in agreement with the laboratory observations on fracturing process (Mogi 1985).

In respect to the previous studies (Rossi 1990, 1994). mainly focused on the seismic sequence analysis, the present one considers the seismic activity with the aim of:

1. Determining whether the seismicity has a fractal character;
2. Using the fractal dimension as quantitative information on the shape of the seismicity spatial distribution: more in detail, inquiring whether its volumetric distribution is mainly linear, planar or tending to fill the whole. In fact, if the hypocentres propagate in one particular direction or align along a line on a fault plane, the relative fractal dimension will be near to 1. If they distribute on a plane, *D* will be 2 or near to 2. If the earthquakes distribute on different lines on the plane, the relative fractal dimension will be greater than 1 and smaller than 2; the nearer to this number, the more are the lines and the intersections. Analogously, a strong clustering will give a *D* near to 0 whereas *a D* greater than 2 and near to 3 indicates the tendency to fill a volume and, therefore, the plausible activation of many planes, with various orientations.

**Fig. 9:**
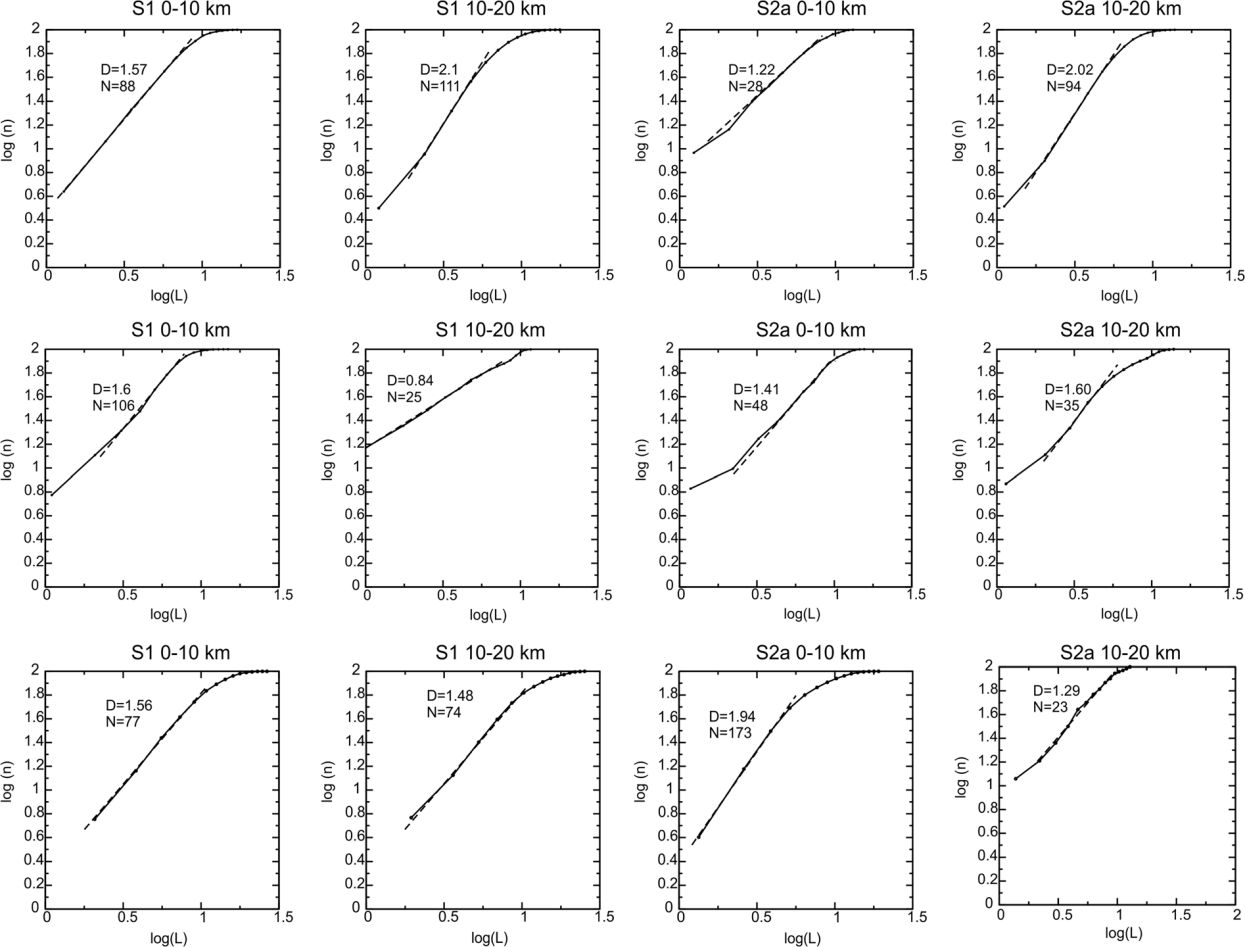
Correlation integral curves for the 12 data sets corresponding to the two depth intervals for the cross sections S1, S2, S3, S5 and S9. The indication of the polygon, as well of the depth interval considered, is reported above the diagram, the resulting fractal dimension (*D*) and the event number (*N*) in the diagram itself. The *dashed lines* indicate the linear regression

To achieve these goals, we extracted 12 subsets from the declustered catalogue, considering the above-described polygons centred on sections S1, S2, S3 and S9, respectively. Moreover, each subset was separately analysed while further discriminating the two depth intervals of 0–10 and 10–20 km depths. Unfortunately, this implies a variable earthquake number over which to calculate the fractal dimension. The maximum earthquake number (173) characterizes the shallowest depth interval of section S9, followed by S1 with 88 and 111 in the two depth intervals considered. The minimum earthquake number is in S9 and polygon S2b, both for the 10–20-km depth interval, with 23 and 25 earthquakes, respectively). Twenty-eight events are in S2a (0–10-km depth) and 35 in S3 (10–20-km depth). Following the above criteria, we gave the score A, B and C to our data sets. The results are summarized in Fig. 9 and Table 1.

**Table 1:** Fractal dimension (spatial correlation integral) for the polygons centred on the sections S1 S2, S3, S5 and S9 of Fig. 11

Section	Depth interval (km)	N. events	Fractal dimension (*D*_*c*_)	Linear fit (RMS)	Quality
S1	0–10	88	1.57	3.00 × 10^−06^	A
S1	10–20	111	2.10	1.32 × 10^−05^	A
S2a	0–10	28	1.22	4.30 × 10^−04^	B
S2a	10–20	94	2.02	1.76 × 10^−06^	A
S2b	0–10	106	1.61	1.40 × 10^−04^	A
S2b	10–20	25	0.84	5.40 × 10^−05^	C
S3	0–10	48	1.41	9.60 × 10^−05^	A
S3	10–20	35	1.59	1.50 × 10^−04^	A
S5	0–10	77	1.56	1.05 × 10^−04^	A
S5	10–20	74	1.48	4.00 × 10^−08^	A
S9	0–10	173	1.94	4.04 × 10^−05^	A
S9	10–20	23	1.30	2.85 × 10^−04^	C

For each depth interval, the earthquake number for each subset (N. Events), the fractal dimension (*D*_*c*_), the RMS of the linear fit and the quality of the analysis are indicated

The curves in Fig. 9 show a general linear trend on a log-log diagram. Slight deviations from the linearity are observed for S2a (0–10-km depth) and S3 and S9 (10–20-km depth). This can be due to a limited event number and consequent lack of observations at all the distance intervals. According to our criteria, however, the sole S9 (10–20-km depth) data set has quality C, whereas S2a (0–10-km depth) has quality B and S3 (10–20-km depth), even A. The RMS errors of the linear best fit are small, varying from the minimum of 4.0 × 10^−8^ of S5 (10–20-km depth) to the maximum of 4.3 × 10^−4^ of S2a (0–10-km depth) (Table 1).

In the analysed cases, the fractal dimension ranges from the minimum of 0.84 of S2b (10–20-km depth) to the maximum of 2.1 of S1 (10–20-km depth). Hence, in three cases, the hypocentres show *D*_*c*_ ≈ 2 and hence the tendency to fill a plane: S1 (10–20-km depth), S2a (10–20-km depth) and S9 (0–10-km depth). Distributions with *D*_*c*_ ≈ 1, characteristic of a linear distribution, are found for S2a (0–10-km depth) and S9 (10–20-km depths). The fractal dimension smaller than 1 of S2b (10–20-km depths) indicates a strong clustering of the shocks, also confirmed by the high number of couples at a short distance of the diagram of Fig. 9. It is, however, noteworthy that S2a (0–10-km depth), S9 (10–20-km depth) and S2b (10–20-km depth) have the smallest shock number (<30) and have been evaluated of lesser reliability (B and C), for the possible incomplete sampling of the distances.

In all the other cases, fractal dimension lies in the interval 1.41 ≤ *D*_*c*_ ≤ 1.61.

We can interpret these values as indicative of a distribution that tends to the planarity, without reaching it. This can mean that the earthquakes distribute on a plane following one or more preferential propagation directions or, even, that linked fault segments are activated, following a dominant trend. The following analysis is aimed to try to discriminate between these two possibilities.

## Principal component analysis

Fractal dimension describes the hypocentre spatial distribution in terms of planar or linear structures, but it does not tell us anything about their orientation, necessary for a comparison with the known tectonic lineaments of each area. The principal component analysis (PCA) of the spatial distribution of the same hypocentres can, on the contrary, provide such information.

PCA, also known as principal parameters method, empirical orthogonal functions (EOF) method or Karhunen-Loève transform, is a dimension-reduction method utilizing the first (centroid) and second moments (variance) of the measured data. It is widely used among others in engineering, meteorology, biology, social sciences and image recognition. Its popularity is due to the capability of retrieving common features from various observations, through the singular value decomposition of the covariance matrix. As a result, from an original *n*-dimensional data set, we obtain a concise description of it in terms of *m* orthogonal functions (*m < n*), accounting for the variance of the data set. Pearson (1901). in one of the first descriptions of the method, underlined its capability of identifying the best fitting plane to a system of points as the plane normal to the least axis of the correlation ellipsoid. The mean square residual is proportional to the length of the least axis itself.

As regards the application of this method to seismology, it proved to be powerful in revealing the orientation of planar features from the spatial distribution of hypocentres, ordered in time by sliding a time window of fixed width along the sequence of events. The obtained correlation ellipsoid is also named rupture ellipsoid (Ebblin and Michelini 1986; Michelini and Bolt 1986; Tselentis et al. 1989; Bressan et al. 2009).

Rossi and Ebblin (1990) extended the method to a multidimensional approach, including time as an independent variable, to evaluate the hypocentre distribution in the space-time domain. The shape and volume of the so-obtained rupture hyper-ellipsoid enable to evidence the onset of earthquake bursts related to the activation of a single fault set. The hyper-ellipsoid axes projection into a 3D space depicts the fracture orientation, as for the 3D case. Moreover, the fourth, namely the time axis, indicates the fracture propagation direction and its variations over time as the sequence evolves (Rossi and Ebblin 1990).

Both in 3D as well as in 4D, the set of components or parameters ***C*** *=* [*c*_*ij*_] are defined as

9 $$ {c}_{ij}={c}_{ji}={N}^{-1}{\displaystyle \sum_{k=1}^{k=N}\left({x}_{ki}-\overline{x_i}\right)\left({x}_{kj}-\overline{x_j}\right)} $$

where *N* is the earthquake number, *x*_*ki*_ and *x*_*kj*_ are the hypocentre coordinates in the *nd*-dimensional space, for *i*, *j* = 1,.., *nd* and

10 $$ \overline{x_i}={N}^{-1}\sum_{k=1}^{k=N}{x}_{ki} $$

for *i* = 1,.., *nd* are the coordinates of the foci centroid.

The matrix ***C*** can be represented by a *nd*-dimensional hyper-ellipsoid. Its principal semi-axes can be obtained through the singular value decomposition from the eigenvalue square roots *R*_*i*_ of the matrix ***C***, with *i* = 1,.., *nd,* for the length, and from its eigenvectors *u*_*i,j*_ (*i*, *j* = 1,.., *nd*) for the orientation.

In the present work, we aim to investigate whether the seismicity tends to locate on a preferential plane. For this reason, we preferred the 3D approach in respect to the 4D one, since the correlation of the spatial dimension with the time-dimension appears weaker than in the case of sequence analysis. Hence, the best-fit plane of the hypocentre distribution is indicated by the first two ellipsoid’s axes. The mean square residual (SR) is proportional to the minimum axis, as stated by Pearson (1901):

11 $$ SR=\sqrt{\varDelta}\cdot {R}_{min} $$

where Δ is the matrix determinant and *R*_*min*_ is the ellipsoid’s minimum axis.

By analysing the seismic sequences, we can reasonably assume that the time-ordered hypocentre spatial pattern is representative of the main rupture plane. This is not necessarily true when treating a declustered catalogue, also in spatially limited areas. In fact, in time, related to the fault growth and propagation, various secondary faults and splays are activated in the volume surrounding the main fault. The best-fit plane of the hypocentre distribution, therefore, in such cases, will represent the mean or most representative orientation, and the minor axis, the width of the faulted and damaged zone.

### Seismic data PCA analysis

The principal component analysis was already applied to the study of seismicity characteristics in Friuli (Ebblin and Michelini 1986; Michelini and Bolt 1986; Rossi and Ebblin 1990; Bressan et al. 2009). In all cases, however, the method was applied to individual seismic sequences, i.e. when the earthquakes are expected to delineate in time the fracture plane. We apply the PCA analysis to investigate whether the seismicity over a time interval of 25 years locates on preferential planes.

From the fractal analysis, we observed that only in a few cases, the fractal dimension shows that the hypocentres clearly define a plane. It is the case of subsets S1 (10–20-km depth), S2a (10–20-km depth) and S9 (0–10-km depth). In the other cases, the fractal dimension indicates a distribution intermediate between a line and a plane. We can interpret such a result as due to a partial filling of the plane, or vice versa as the tendency of earthquakes to migrate in a particular direction. The aim of the application of PCA analysis to the same subsets is to determine the orientation of both planes and linear alignment of hypocentres, for a possible interpretation in terms of tectonic domains and structures.

The time sequence is maintained for each subset described above, by sliding along the temporal sequence of events a time window of fixed earthquake number for the calculus of the correlation ellipsoid.

**Fig. 10:**
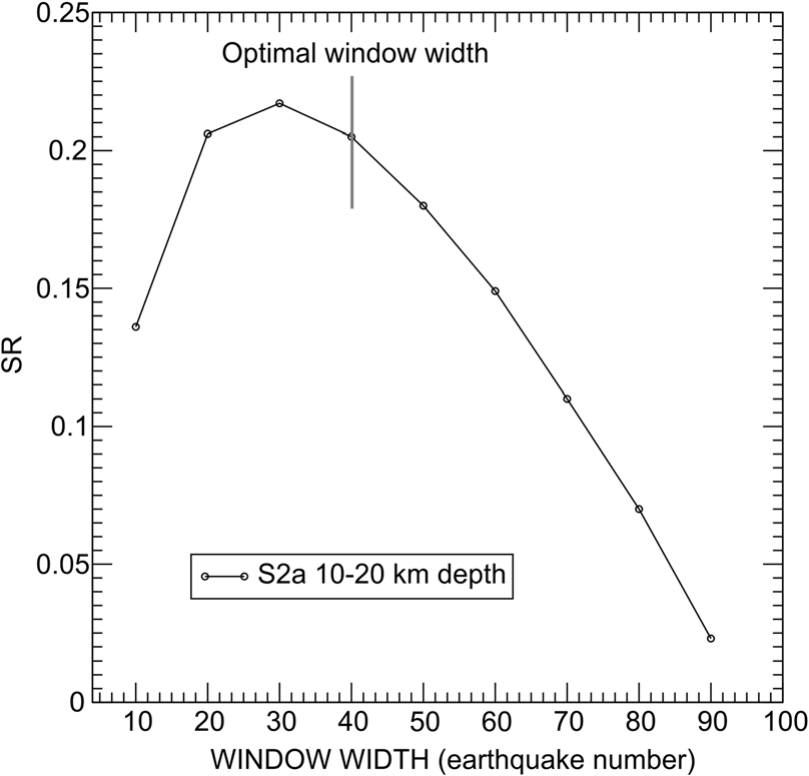
Subset S2a, depth interval 10–20 km. Variation of the square mean residual ($SR=\sqrt{\varDelta}\cdot {R}_{min}$, where Δ is the matrix determinant and *R*_*min*_ is the minimum ellipsoid axis, of the best fitting plane for different time window widths. *Y*-axis: square mean residual (*SR*); *X*-axis: time window width (earthquake number). The optimal width is the one for which *SR* starts to decrease

The optimal window width is chosen by calculating the mean square residual (SR) of the best fitting plane (Fig. 10). After an initial increase, SR begins to decrease steadily as more earthquakes are included in the PCA analysis. We chose as the optimal one the window for which SR starts to decrease, indicating that the related ellipsoid is representative of the distribution of the earthquakes included in the time window.

**Fig. 11:**
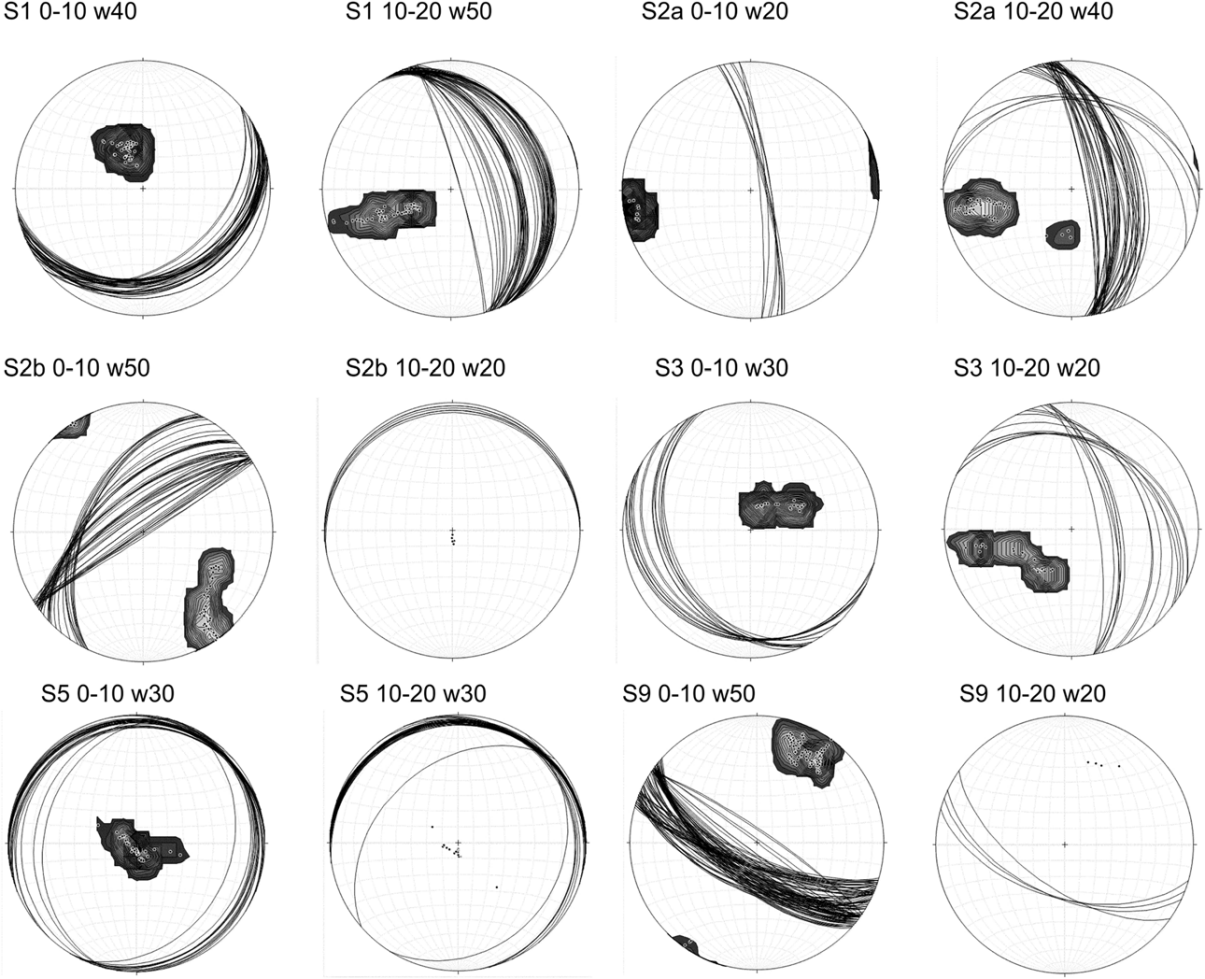
Lower-hemisphere, equal-area projection of the ellipsoid minimum axis (also contoured) for the 12 data sets corresponding to the two depth intervals for the cross sections S1, S2, S3, S5 and S9 of Table 2, and the planes perpendicular to it (*black lines*). The indication of the cross section, the depth interval considered and the window used for the analysis are reported above the stereonet

Figure 11 shows the analysis results, in terms of the ellipsoid minimum axis (*R*_*min*_) directions and of the planes normal to them. Some of the diagrams show two main directions, clearly separated as in S2a (10–20-km depth), S2b (0–10-km depth) and S3 (10–20-km depth). In other cases, the two directions are very near to each other, as for S1 and S3, the first for the depth interval 10–20 km, the second for 0–10-km depth. In Table 2, the trend and plunge of the average mean vector (Fisher 1953) of the *R*_min_ axis for all the polygons are reported together with the confidence limit *α*_95._ The latter is a measure of the precision with which the true mean direction has been estimated (Allmendinger et al. 2012). The smallest value of *α*_95_ is associated with a distribution of planes almost coaxial, as for the case of S1 (0–10-km depth), where the planes vary from N50°E, 28° dip to N80°E, 24° dip. A small *α*_95_ also characterizes S2b and S5 (10–20-km depth), since most of the planes show a strike near to N279°E, 5–6° dip (i.e. Alpine), even if other lineaments are present in the latter case, almost all subhorizontal. Also for S2a (0–10-km depth), the solution is well constrained since the few data give as a solution an almost vertical plane, striking about N350°E, 80° dip. Slightly higher is the *α*_95_ value for S9 (0–10-km depth): it corresponds to a large number of planes with a strike comprised between N106°E and N128°E and dip ranging from 55° to 77° to become almost vertical. The few planes of S9 (10–20-km depth) are consistent with the shallowest data set, all in agreement with the Dinaric structures present in the area. In the remaining cases, two main directions are recognizable, with a gradual rotation of the axes in the case of S1 (10–20-km depth), S2b (0–10-km depth) and S3 (0–10-km depth) and a sudden change, on the contrary, for S2a and S3 (10–20-km depth). For S1, we pass from N328°E, 23° dip to N343°E, 84° dip (i.e. Dinaric), whereas for S3 (0–10-km depth), the rotation is from N159°E, 34° dip to N105°E, 15° dip, and for S2b from N235°E, 90° dip to N205°E, 55° dip. As said, for S2a and S3 (10–20-km depth), two orientations are present, with a similar pattern: a first family of planes with strike about N151°E, with dip varying from 55° to 77°, better represented in S2a, and another, from N295°E to N308°E and dip from 27° to 35°, better represented in S3.

**Table 2:** Ellipsoid *R*_*min*_ average trend and plunge for the polygons centred on the sections S1, S2, S3, S5 and S9 of Fig. 11, resulting from the calculus of the *R*_*min*_ distribution

Section	Depth interval (km)	N. events	*R*_min_ trend	*R*_min_ plunge	*α*_95_
S1	0–10	88	337	63	1.6
S1	10–20	111	247	50	3.6
S2a	0–10	28	259	11	2.8
S2a	10–20	94	256	31	4.3
S2b	0–10	106	137	24	8.5
S2b	10–20	25	176	84	2.0
S3	0–10	48	49	67	4.7
S3	10–20	35	245	45	9.9
S5	0–10	77	191	86	2.4
S5	10–20	74	187	84	1.8
S9	0–10	173	30	21	3.2
S9	10–20	23	24	32	8.7

The average direction and its confidence limit *α*_95_, a measure of the precision with which the true mean direction has been estimated, are calculated according to Fisher statistics (Fisher 1953; Allmendinger et al. 2012)

## Discussion

The comparison of crack density and seismicity spatial patterns suggests that the earthquake occurrence is related to material heterogeneities in the depth interval 0–10 km. The seismicity appears mainly distributed in areas characterized by variation from low (0.1) to intermediate (0.4) crack density values, except for few limited zones at 8-km depth where some events are localized into low crack density (less than 0.1) areas (Fig. 6a–c). The crack density pattern is consistent with the elastic moduli pattern of the study area upper crust, obtained with the sequential integrated inversion of tomographic velocity images and gravity data (Bressan et al. 2012).

This result was not a foregone conclusion because we introduced a novelty in the model formulated by Zhao and Mizuno (1999). They applied the OB74 theory assuming that uncracked rocks have the maximum seismic velocity inverted from seismic tomography at each depth in the study area. In our approach, the elastic moduli of the cracked rocks are represented by the values of the elastic moduli obtained from the integrated inversion of tomographic images and gravity data. The novelty is that the elastic moduli of the uncracked rocks are represented by the values of the elastic moduli obtained from laboratory measurements at 400 MPa confining pressure (Faccenda et al. 2007). on the samples of the most representative lithologies of the sedimentary rocks of the upper crust (0–10-km depth). The lithotypes were assigned according to the geological cross sections.

Hence, low *ε* areas are consistent with the areas characterized by the in situ high rigidity (shear modulus) and vice versa. The high-rigidity bodies, characterized by low crack density, are surrounded by sharp variations of the elastic moduli and by increasing crack density. It should be noted that the pattern of damage, expressed in a classic relation between effective elastic modulus *G*_*eff*_ and elastic modulus of damaged materials *G*_*D*_, is very similar to the crack density pattern.

Our interpretation is based on the arguments put forward by Maxwell and Young (1992) and Koulakov et al. (2010). The areas with high crack density correspond to highly fractured rocks that cannot store high strain energy. Low crack density zones represent more competent and poorly fractured zones that are capable of sustaining higher strain energy. The strain stored is likely released in areas with a sharp transition from low to higher crack densities, favouring brittle failure.

If a mechanically heterogeneous body is subject to a stress field, the interface separating media with different elastic moduli is energetically favourable for the localization of fractures.

Chatterjee and Mukhopadhyay (2002) investigated the interactive effect of applied stress and the variations of rock mechanical properties. They found that stress concentration is high and that the maximum rotation of the principal stresses occurs in zones, where the contrast in elastic properties of rocks is the greatest. The zones of marked variation of the elastic properties of rocks modify the stress field by relaxation processes, through the variation of the effective confining pressure and the rotation of the principal axes of stress. The rotation and variations (expansions and contraction) of the stress tensor can reduce the fracture normal stress on the fracture surfaces, favouring the occurrence of rock failure.

The areas with a high saturation rate are partially consistent with those of high crack density. The discrepancies can be due to the limits of the OB74 model, represented by the applicability of the crack model in cases with large crack density and relevant variation of mechanical properties. Takei (2002) claimed that the OB74 model is less accurate in cases of high crack density and different pore geometry. In addition, the OB74 model assumes that microcracks are randomly distributed in the volume rock. This assumption is not valid in cases where microcracks are oriented along particular directions induced by tectonic stress field (Zhao and Mizuno 1999). as probably is our case.

**Fig. 12:**
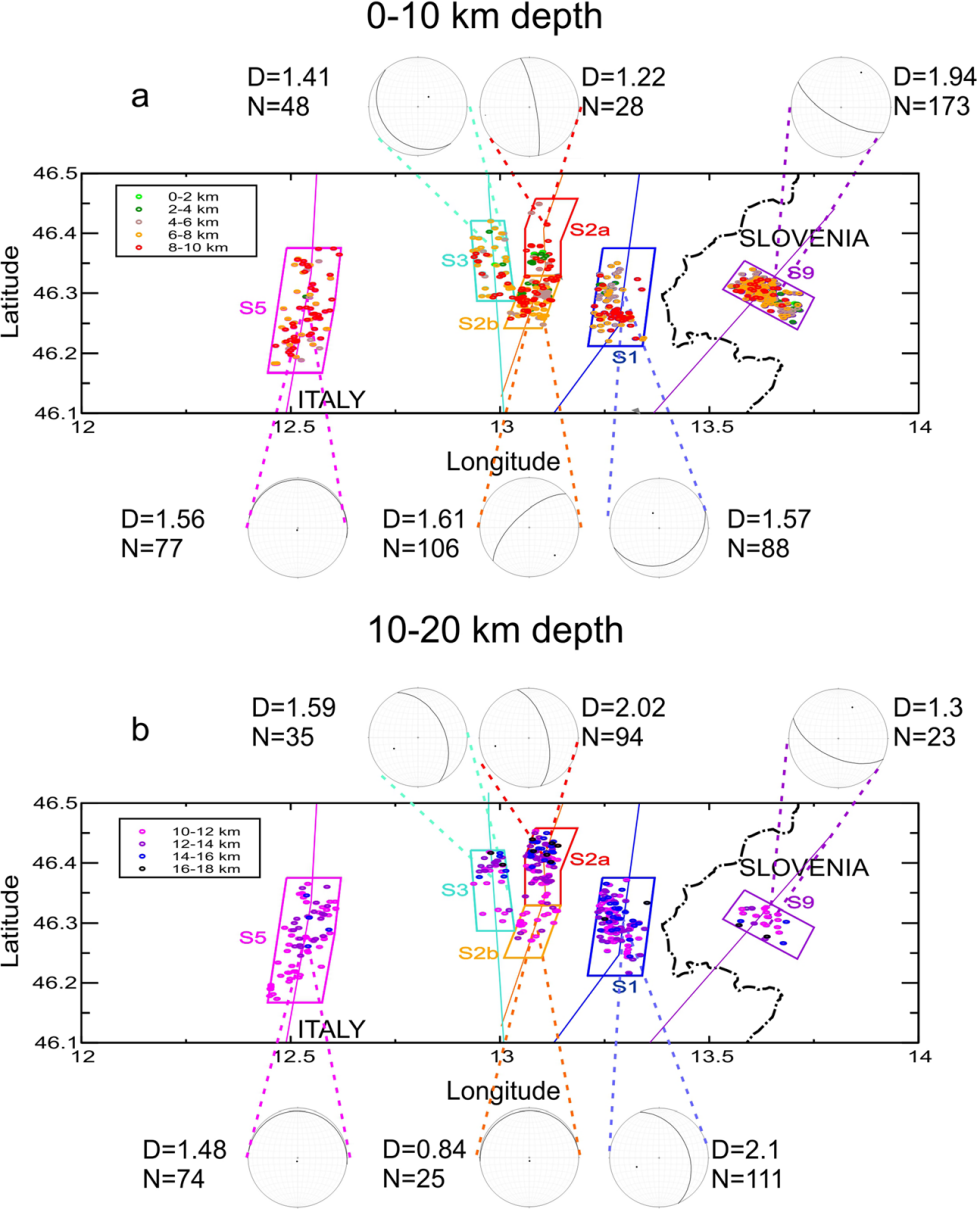
Hypocentre geometrical analysis results for the depth interval 0–10 km (**a**) and 10–20 km (**b**). The horizontal projections of the *boxes* traced across the sections S1, S2, S3, S5 and S9 are drawn, together with the hypocentres of each subset. Two-kilometre depth intervals are marked with *different colours* (see *boxes*). The stereograms with the mean solution from Fig. 10 are reported in correspondence of the relative section, as well as the fractal dimension (*D*) and the earthquake number (*N*)

Figure 12 summarizes the results of the geometrical analyses done for the 12 seismic subsets: the fractal and the PCA analysis. In the latter case, the stereogram shows the average plane, i.e. normal to *R*_*min*_ (Table 2). The six polygon traces, with the relative projected hypocentres, are also shown. The depth intervals of the hypocentres are indicated with different colours: from green to red in the shallowest depth interval and from magenta to black in the deepest one. The most evident feature is the consistency of the solution for the S9 polygon at two depth intervals, notwithstanding the difference in earthquake number (173 earthquakes in the first depth range, and only 23 in the second one). It depicts clearly a sub-vertical plane (strike N122°E, 75° dip), coherent with the orientation of the Dinaric lineaments present in the area and in particular with the Ravne Fault, where the 1998 and 2004 Bovec-Krn (W Slovenia) seismic sequences are located (Bajc et al. 2001; Bressan et al. 2009; Kastelic et al. 2008). The fractal dimension of 1.94 of the shallow part of the subset, characterized by the highest earthquake number, confirms the tendency of the hypocentres to fill a plane. The value 1.3 of the deepest part is less reliable, being the quality evaluated as C. The other feature emerging from Fig. 11 is the greater homogeneity of the solutions in the deepest part (10–20-km depth), with respect to the shallowest one (0–10-km depth).

Between 10- and 20-km depths, in fact, S1, S2a and S3 show planes with a strike from N329°E to N342°E dipping 29° for S1, and about 45° for both S2a and S3, concordant with the Dinaric lineaments. In all the cases, the tendency to fill the plane is confirmed, being *D*_*c*_ = 2.1, *D*_*c*_ = 2.02 and *D*_*c*_ = 1.6, respectively. We recall that S1, S2a and S3 in this depth interval are characterized by multiple solutions (Fig. 10), but the Dinaric NW-SE trend appears to dominate. The distribution in depth, evidenced by increasingly dark colours, is in agreement with the dip of the planes from PCA solution. S2b and S5, despite the different hypocentres number, also show coherent solutions, subhorizontal (dip 5–7°) and E-W trending, in agreement with the Alpine lineaments present in the area (Ponton 2010). As already said, the small fractal dimension value of S2b is poorly constrained, due to the limited number of earthquakes (quality C). In the S5 case, the value 1.48 can indicate that the earthquake distribution does not fill a whole plane. The depth distribution, with hypocentres mainly in the 10–12-km depth interval, agrees with a subhorizontal plane.

The shallowest depth interval shows a higher variability. Excepting the already mentioned S9, we find NE-SW orientations (anti-Dinaric), but with different dips, in S2b (N227°E, dip 66°) and S1 (N66°E, dip 24°), an about N-S trend for S2a (N355°E, dip 82°) and a Dinaric orientation for S3 (N128°E, dip 21°). S5 shows an Alpine E-W trending, subhorizontal, and similar to the deepest part. In this depth interval, fractal dimension lies in the interval 1.41 ≤ *D*_*c*_ ≤ 1.61, with the exceptions S9 (*D*_*c*_ = 1.94) and S2a (*D*_*c*_ = 1.22). In this last case, the earthquake spatial distribution of Fig. 12 suggests that there is the tendency of earthquakes to propagate from one of the other extremes of the polygon, in agreement with a *D*_*c*_ close to 1 and to the orientation of the plane inferred from PCA, about NS. We remind, however, that this data set has been evaluated of poor reliability (quality C) for the possible incomplete sampling of the distances. The depth distribution of the hypocentres confirms a nearly vertical distribution of them.

Values comprised between 1 and 2 are indicative of a distribution that tends to the planarity, without reaching it. Analysing the results of Fig. 12, in the case of S1, it appears that the hypocentres distribute on a planar surface, without filling it totally. In the other cases, often showing multiple solutions, with different dips and a gradual transition from one solution to another (Fig. 11), we can hypothesize that linked fault segments are activated.

We also remind that, by analysing a declustered catalogue, we are inferring the mean or dominant orientation of the main faults and their splays that activated in time during the 25 years of the catalogue. This can explain the discrepancy between the dip of our average planes and the dip of the few known outcropping fault planes, while the strike is coherent. On the other hand, in limited areas, as for our S9, the PCA analysis performed by using a temporal sliding window of appropriate length can be used to follow the growth and evolution of fracturing. The multiple solutions found in our analysis can reflect, in cases as Ravne Fault, the activation of splays, Riedel shears, conjugate planes or the activation of pre-existing discontinuities that characterize a fault and the damaged volume. By summarizing, the area characterized by the maximum interference pattern between the Dinaric and the Alpine fault systems is affected by the highest seismic activity. The PCA analysis of the seismicity evidences different patterns in the two depth intervals here considered. In the 10–20-km depth range, the seismicity is mainly located on the Dinaric lineaments in the northern and eastern parts of the study area, while on Alpine thrusts in the western and southern parts. In the shallow depth interval (0–10 km), on the contrary, the solutions appear more variable, but a coherence with the tectonic structures present in the area can be found, with anti-Dinaric and Alpine trends. The other striking example is given by the seismicity pattern featuring the sub-vertical strike-slip Ravne Fault (Fig. 12).

The area of the maximum interference pattern between the Dinaric and the Alpine tectonic structures also corresponds to areas of sharp variations of the crack density pattern (Fig. 6). In our interpretation, we relate the occurrence of seismicity in the shallowest crust to the mechanical rock heterogeneities. At depths greater than 10 km, on the contrary, the influence of Dinaric and Alpine lineaments on the seismicity distribution appears more distinct.

The obtained fractal dimension values indicate that the hypocentre pattern has a dominant propagation along fracturing lineaments, filling only partially a plane. The seismicity apparently tends to fill the plane only in three cases, all with Dinaric trend.

We, therefore, conclude that the effects of the different tectonic phases are recognizable in the depth interval 10–20 km. In the shallowest part of the region, on the contrary, the pattern of seismicity appears mainly conditioned by mechanical discontinuities, highly variable pore pressure and lesser confining pressure.

## Conclusions

Two non-conventional methods of analysis of the spatial organization of seismicity are used to investigate the region across NE Italy and W Slovenia: fractal analysis and PCA analysis. The fractal analysis helps to discriminate the cases in which hypocentres clearly define a plane, from the ones in which hypocentre distribution tends to the planarity, without reaching it. The PCA analysis is used to infer the orientation of planes fitting through earthquake foci. Moreover, in the cases of fractal dimension lower than 2, PCA analysis, performed with sliding temporal window, helps to understand whether earthquakes distribute on a plane following one preferential propagation direction or they are due to the activation of linked fault segments in the fault growth process.

Furthermore, the spatial seismicity pattern at the shallow depths is investigated in the context of general damage model, represented by the crack density distribution, following and improving the method of Zhao and Mizuno (1999).

The fractal analysis evidenced that the hypocentre pattern, in general, fills only partially a plane. The most frequent values of the fractal dimension, in fact, lay within 1 and 2. In some cases, there is a clear tendency of the events to propagate in a particular direction. It appears, however, that linked fault segments are activated in time in the majority of the cases. The seismicity spatial distribution fills a plane only in three cases, all with Dinaric trend, without interference with Alpine lineaments. The PCA analysis provides different patterns in the two depth intervals here considered. The seismicity tends to define planes with variable orientations mainly in the shallow depth range (0–10 km), characterized by a significant interference pattern between the Dinaric and the Alpine tectonic structures. The spatial seismicity patterns appear closely related to the variation of the mechanical properties of the crust. The seismicity is mostly distributed in areas characterized by a variation from low to intermediate crack density, corresponding to the sharp transition from zones of low damage to zones of intermediate damage. Low crack density zones correspond to more competent rocks capable of sustaining high strain energy, while high crack density areas are pertaining to intensively fractured rocks that cannot store high strain energy. Brittle failure, and hence the seismicity, is favoured within the sharp transitions from low to intermediate crack density zones. In fact, the orientation of the planes depicting the seismic activity coincides with the orientation of the faults generated along the flanks of the former carbonatic platforms both in Friuli and western Slovenia.

The 10–20-km depth interval is characterized by a more organized pattern of seismicity. The latter is mainly located on the Dinaric lineaments in the northern and eastern parts of the study area while on Alpine thrusts in the western and southern parts.
